# Anisotropic Response of CoCrFeMnNi High-Entropy Alloy Fabricated by Selective Laser Melting

**DOI:** 10.3390/ma13245687

**Published:** 2020-12-13

**Authors:** Bowen Wang, Miao Sun, Bobo Li, Lijuan Zhang, Bingheng Lu

**Affiliations:** 1 State Key Laboratory of Manufacturing System Engineering, Xi’an Jiaotong University, No. 99 Yanxiang Road, Xi’an 710054, China; wangbowen@stu.xjtu.edu.cn (B.W.); sunmiao@niiam.cn (M.S.); boboli@stu.xjtu.edu.cn (B.L.); 2 School of Mechanical Engineering, Xi’an Jiaotong University, No. 28, Xianning West Road, Xi’an 710049, China; 3 National Innovation Institute of Additive Manufacturing, No. 997, Shanglinyuan 8th Road, Gaoxin District, Xi’an 710300, China

**Keywords:** selective laser melting, high-entropy alloy, anisotropy, microstructure, mechanical properties, corrosion

## Abstract

This study investigated the anisotropic characteristics of the microstructural, mechanical and corrosion properties of CoCrFeMnNi high-entropy alloy produced by selective laser melting (SLM) additive manufacturing (AM). Under the extremely high thermal gradient during the SLM process, a columnar solidification structure with a single face-centered cubic (FCC) phase structure was formed. The crystal structure exhibited a regular checkerboard structure in the XOY plane (perpendicular to the building direction), which was composed of {110} direction and a small amount of {100} fiber texture. The cellular-dendritic sub-structures formed in the columnar crystal structure with sizes of about 500 nm in diameter. As for the mechanical properties, the XOY plane exhibited higher ultimate tensile strength and yield strength (σ_0.2_) but lower elongation to failure compared to the XOZ plane (parallel to building direction), which reflected the anisotropy of the microstructure. The electrochemical test results of the different planes showed that the XOZ plane exhibited better corrosion resistance in comparison with the XOY plane in the 3.5 wt % NaCl solution, which was on account of the selective attack at the Mn-rich inter-cellular regions and the different structures of the cellular-dendritic sub-structures on different planes.

## 1. Introduction

Selective laser melting (SLM) is one of the additive manufacturing technologies that has been recognized as an affordable and efficient solution to manufacture complex metal parts with near full density. In SLM processing, a controllable intensive laser beam was used to selectively fully melt and solidify the metal powder bed in a layer-by-layer manner according to the scanning strategy provided by the sliced computer aided design (CAD) file. The unique building method and relatively high cooling rate of SLM made it possible to manufacture parts with complex shapes and form unique microstructures to produce excellent mechanical properties, which could not be easily obtained by traditional methods [1,2,3].

As a new class of metallic materials, high entropy alloys (HEAs) have spurred great interest since they were proposed [4,5]. In contrast to traditional alloy design strategy, high-entropy alloys consist of five or more multiple principle elements mixed in an equiatomic or near-equiatomic ratio. Because of their superior mechanical, physical and chemical properties, they are considered as an alternative for traditional metallic materials [6]. As a typical HEA, the CoCrFeMnNi single-phase solid solution with a face-centered cubic (FCC) crystal structure has been widely studied owing to its excellent mechanical properties, especially the combination of high strength and ductility at high and low temperatures [7]. Some works have been conducted on the CoCrFeMnNi fabricated by additive manufacturing. Piglione et al. [8] produced single-layer and multi-layer builds by SLM to investigate the microstructure and crystal orientation by scanning electron microscopy (SEM) and electron backscatter diffraction (EBSD). It was found that CoCrFeMnNi had excellent printability and had uniform high hardness. Li et al. [9] studied the effect of laser energy density on the densification of the CoCrFeMnNi high-entropy alloy, and fabricated the bulk high-entropy alloy at the parameters with highest density. The non-equilibrium solidification microstructure and mechanical properties of SLM produced high-entropy alloy were studied. The results showed that the density and mechanical properties of SLM produced CoCrFeMnNi high-entropy alloy were improved by hot isostatic pressing (HIP). Chen et al. [10] used the SLM method to prepare CoCrFeMnNi high-entropy alloy by in-situ alloying of CoCrFeNi pre-alloyed powder and Mn element powder. The results showed that the mixed powder had high printability. Despite the slight evaporation of Mn at the high energy density, it had uniform elemental distribution. Kim et al. [11] fabricated the equiatomic CoCrFeMnNi high-entropy alloy successfully by SLM method. The compression properties in different building directions were tested, and the relationship between the microstructure, mechanical properties and deformation mechanism were discussed. Chew et al. [12] investigated the microstructure and mechanical behaviors of the laser aided additive manufacturing (LAAM) fabricated HEAs. Compared with the conventionally cast HEAs, the CoCrFeNiMn fabricated using LAAM possessed significantly higher yield strength and ultimate tensile strength, because of the finer grains. Ren et al. [13] and Xu et al. [14] comparatively analyzed the corrosion behavior of the SLM-produced and as-cast CoCrFeMnNi HEA in 3.5 wt % NaCl solution. Compared with as cast material, CoCrFeMnNi high-entropy alloy prepared by SLM had better composition uniformity and better corrosion resistance. So far, laser additive manufacturing built CoCrFeMnNi alloys showed more excellent mechanical properties than the cast alloys. Meanwhile, the SLM-built components tend to have column grains that grew along the high cooling rate direction [15]. The oriented microstructures always lead to anisotropic material properties, such as mechanical properties and electrochemical properties, which had significant effect on practical applications. Therefore, it was necessary to characterize the anisotropic microstructure and properties of SLM-built components systematically and comprehensively.

In this work, equiatomic CoCrFeMnNi HEA specimens were prepared by SLM using optimized parameters through a combined approach. The mechanical properties and electrochemical properties taken in longitudinal and transverse direction (with respect to the build direction) of the SLM processed CoCrFeMnNi alloys were characterized. The results presented here provided a factual basis for the future application of SLM-built CoCrFeMnNi alloys.

## 2. Materials and Methods

### 2.1. Sample Preparation

**Figure 1:**
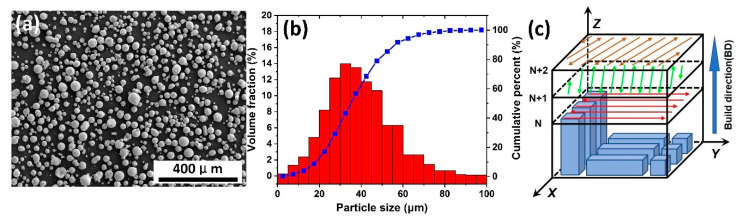
Powder morphology of CoCrFeMnNi alloy (**a**), particle size distribution (**b**) and schematic drawing of selective laser melting (SLM) scan strategy and printed sample orientation (**c**).

The gas atomized equiatomic CoCrFeMnNi HEA powder was used for the specimen preparation in the SLM process, as shown in Figure 1a,b. According to the result of the laser diffraction particle size analyzer (Helos-Rodos), the particle size distribution varied from 15 to 53 μm and the average size was about 38 μm (D10 = 21.02 µm, D50 = 37.52 μm, D90 = 58.70 μm), as shown in Figure 1b with the red bar repeating frequency distribution and blue line standing for cumulative distribution. The scanning electron microscope (SEM) image (Figure 1a) of the powder morphology showed that the powders were mainly spherical and contain some satellite particles. The chemical compositions of the powder and as-built samples were listed in Table 1, which were determined by X-ray fluorescence spectrometer (XRF, Bruker S8 Tiger). All samples were manufactured in a BLT-S200 SLM machine (Xi’an, China), which was equipped with a 400 W fiber laser (1070 nm wave length), with a focal beam diameter of 100 μm. The processing parameters included the scanning speed of 870 mm/s, laser power of 280 W, hatch spacing of 90 μm, and the layer thickness of 40 μm. The zigzag pattern was adopted, which was rotated by 67° between each successive layer to reduce the stress concentration, as shown in Figure 1c. Some cubic specimens with the dimension of 10 × 10× 10 mm^3^ were prepared to study the microstructure and electrochemical corrosion performance, and some rectangular specimens with the dimension of 10 × 10 × 32 mm^3^ were fabricated parallel and vertical to the building direction for tensile tests. The XOY (vertical to building direction (BD)) and XOZ (parallel to building direction (BD)) planes were selected to study the anisotropy of the samples.

### 2.2. Microstructural Characterization

The cubic metallographic specimens were sectioned parallel to the XOY and XOZ planes by using an electrical discharge machine. Then they were ground with SiC abrasive papers in different grits of 400, 800, 1000 and 1500# respectively, and finally polished with 1 µm diamond suspension. In order to reveal more detailed sub-microstructures, the polished specimens were electrochemically etched in the solution of 10% oxalic acid in water, operated at 5 V for 60 s. The X-ray diffraction (XRD, Brucker D8 Advanced A25, Cu K alpha radiation) was used to identify the crystal structure with a scanning step of 0.02°, range from 20° to 100° for 0.2 s counting time. Metallographic structures of the top surface and side surfaces were observed using an optical microscopy (OM, Leica MeF3A) and a scanning electron microscopy (SEM, JEOL JSM-7900F), operating at 5 kV using secondary electron beam. The crystal orientation and grain size were analyzed by electron backscatter diffraction (EBSD, Oxford Nordlys-CMOS detector). The EBSD step size was set to 1 μm, and the smallest grain size that could be recognized was 4 pixels in the area. The data was analyzed by Channel 5 software. In addition to mechanical polishing, the EBSD sample was polished on an ion thinning instrument (Leica EM RES102), at 5 kV with an inclination of 10° for 30 min, to eliminate the stressed layer. Transmission electron microscopy (TEM, JEOL JEM-2100P) were used to observe the detailed sub-microstructure and analyze the chemical composition of the alloys, with an acceleration voltage of 200 kV. The TEM samples were prepared by a twin-jet electro-thinning device (Denmark Struers A/S) each for 45 s at 18 V, in the solution of 90 mL distilled water (H_2_O), mixed with 730 mL ethanol (C_2_H_5_OH), 100 mL butylcellosolve (C_6_H_14_O_2_), and 78 mL perchloric acid (HClO_4_).

### 2.3. Tensile Tests

Dumbbell-shaped mechanical specimens parallel to the XOY and XOZ planes were machined by lathe from the rectangular specimens with the dimension of 10 × 10 × 32 mm^3^ fabricated parallel and vertical to the building direction, having a diameter of 5 mm and a gauge length of 15 mm. The tensile tests were performed on a universal material testing machine (INSTRON 5982) with a crosshead speed of 1 mm/min at room temperature. The fracture surfaces were then observed by SEM.

### 2.4. Electrochemical Measurements

Both the XOY plane and XOZ plane were selected for electrochemical measurements in order to evaluate the corrosion resistance of different orientations. The copper wires were spot welded on the back of samples for better electrical contact. The specimens with a wire were mounted into epoxy resin with an exposed facet of 10 × 10 mm^2^. The surface of each working electrode was ground to 2000# with silicon carbide papers, and then polished with 0.25 μm diamond slurry. Electrochemical tests were performed by using an electrochemical workstation (Parstat 4000A) with the conventional three-electrode cell in a 3.5 wt % NaCl solution at 25 °C. The sample to be measured, the platinum foil and the saturated calomel electrode (SCE) with E = 0.2415 VSHE were used as the working electrode, counter electrode, and reference electrode, respectively. Before potentiodynamic polarization and electrochemical impedance spectroscopy (EIS) measurements, the open current potential (OCP) was recorded for ~6000 s to ensure a steady measurement condition. The EIS was started with the applied AC amplitude of 10 mV, and the frequency was swept from 100 kHz down to 10 mHz. The potentiodynamic polarization curves were conducted by sweeping the potential from −0.2 mV versus the OCP until the current density exceeded 10 mA/cm^2^ with the scan rate of 0.25 mV/s. After the test, the specimens were cleaned ultrasonically and dried immediately. The morphology of the corroded surface of each specimen was observed by SEM.

## 3. Results and Discussion

### 3.1. Phase Identification

**Figure 2:**
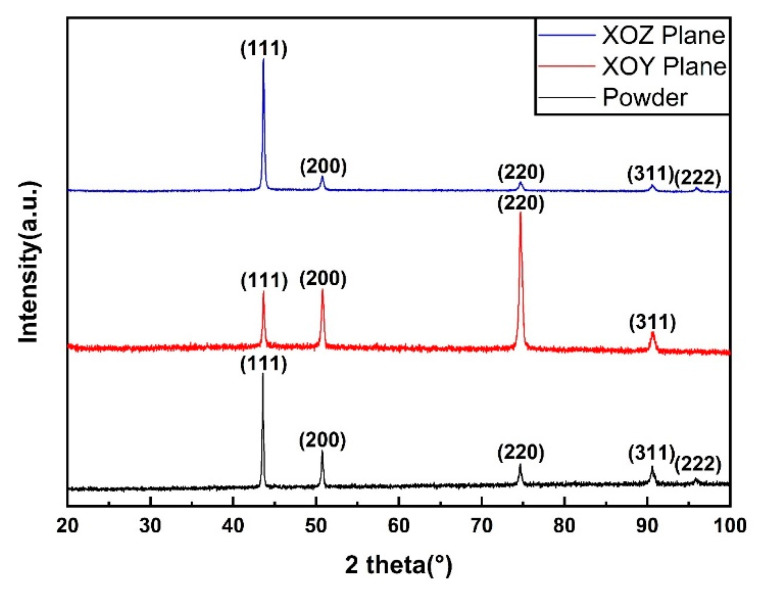
X-ray diffraction patterns of CoCrFeMnNi powder and SLM processed samples.

Typical X-ray diffraction patterns of CoCrFeMnNi powder and as-built specimens of XOY and XOZ planes were shown in Figure 2. There were characteristic diffraction peaks on (111), (200), (220) and (311) planes of FCC crystal detected. The SLMed parts had an identical single face centered cubic phase same as the original powder, although the content of Cr and Mn in the processed parts decreased slightly due to oxidation and burning loss compared with that of the original powder, as shown in Table 1.

Compared with the CoCrFeMnNi powder, the diffraction peak intensities of (200) and (220) of SLM processed samples changed noticeably. In order to make the comparison visible and concrete, the intensity ratio $f_{200}$ and $f_{220}$ were defined to describe the variation.

$$f_{200}=\frac{I_{\left(200\right)}}{I_{\left(111\right)}}$$

$$f_{220}=\frac{I_{\left(220\right)}}{I_{\left(111\right)}}$$

where $I_{\left(200\right)}$, $I_{\left(220\right)}$ and $I_{\left(111\right)}$ represented the diffraction intensities of (200), (220) and (111) respectively and they were the maximum values of the corresponding diffraction peaks read from the Y axis of Figure 2. The values of $f_{200}$ and $f_{220}$ were calculated and listed in Table 2.

Compared with the original powder, both $f_{220}$ and $f_{200}$ values of the XOY plane increased greatly, while for the XOZ plane, both values decreased about 60%, indicating that the relative intensities of (200) and (220) in SLM processed samples were obviously increased on the XOY plane and weakened on the XOZ plane. It implicated that some {110} and {100} fiber texture might exist in the SLM processed samples, which was further analyzed and confirmed in the EBSD section.

### 3.2. Microstructures Characterization

**Figure 3:**
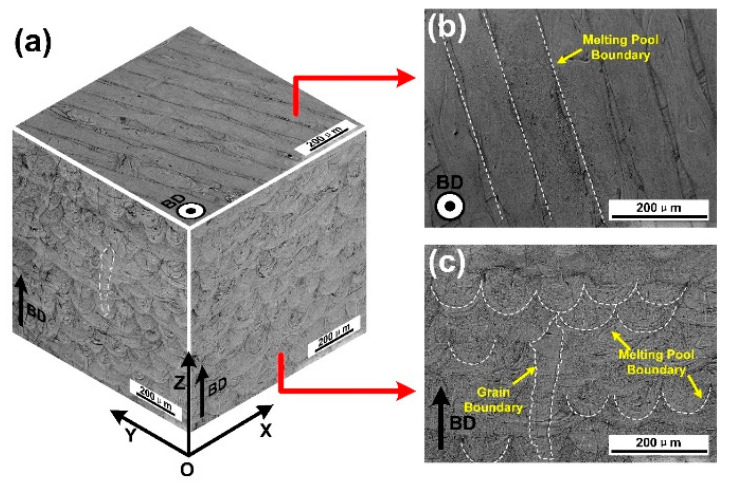
Microstructure demonstration of three-dimensional (**a**), XOY (**b**) and XOZ (**c**) planes of SLM produced CoCrFeMnNi high-entropy alloy taken by optical microscope (BD represents building direction).

Figure 3 shows the three-dimensional morphologies of the CoCrFeMnNi high-entropy alloy prepared by SLM method. Figure 3a is composed of three mutually perpendicular micrographs of the XOY, XOZ and YOZ planes. In order to observe and explain the grain growth and bead building, the enlarged images of XOY and XOZ planes are shown in Figure 3b,c respectively.

The molten pool boundaries on the top surface could be distinguished, marked with white dotted lines. They were parallel to each other owning to the zigzag scanning strategy (the adjacent scanning paths in a layer were parallel to each other with opposite directions) in one layer (Figure 3b). Melt pools with different morphologies were observed in the side view (Figure 3c). In the same side view, the laser molten track of fish scale shape and strip shape could be observed, corresponding to the alternating 67° scanning strategy. In the YOZ and XOZ planes, the width of the molten pool varied from 80 to 100 μm, and the average layer thickness was about 40–50 μm. As shown in Figure 3c, the development of columnar grains microstructures could also be observed, with the grain boundary marked with a white dotted line. These columnar grain size varied 100 to 400 μm in length and 30 to 100 μm in width. They grew parallel to the BD and pass through 4 to 12 layers, which was the result of epitaxial growth. It was known that the growth of columnar grains was always determined by the heat transfer direction along the BD [16].

**Figure 4:**
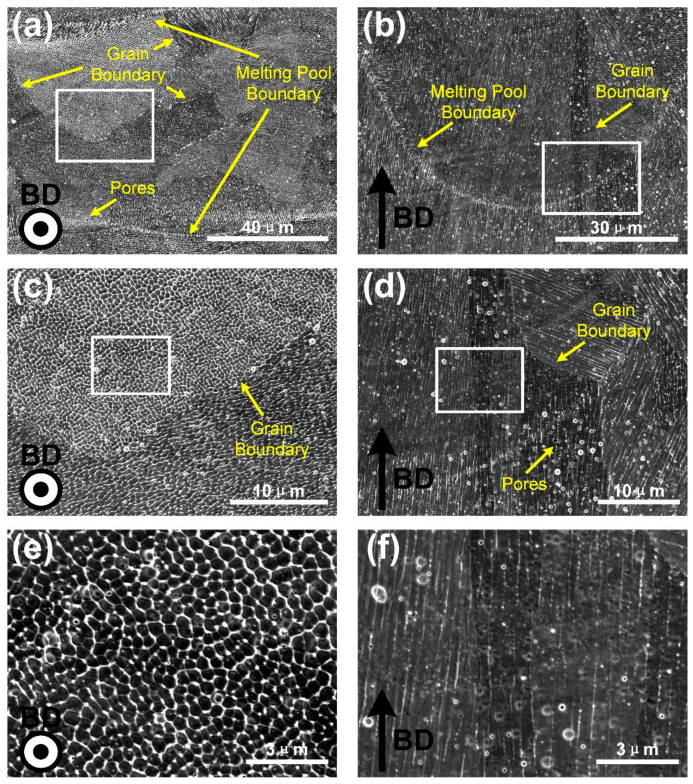
SEM images of top surface XOY plane (**a**,**c**,**e**) and cross section (**b**,**d**,**f**) in different magnifications from a single molten channel of the SLM produced CoCrFeMnNi high-entropy alloy.

In order to further investigate the microstructures of the SLM produced CoCrFeMnNi high-entropy alloy, SEM images of top surface XOY plane and cross section XOZ plane from a single molten channel were taken in different magnifications, as shown in Figure 4. The top surface XOY plane, shown in Figure 4a,c,e, was perpendicular to the BD, and the cross section, shown in Figure 4b,d,f, was parallel to the BD. The microstructures with different scales were revealed, including melting pool boundaries, grain boundaries, cellular sub-structures, and columnar sub-structures, marked with arrows in these micrographs. It could be seen that each grain consisted of many cellular-dendritic sub-structures inside the molten pools, showing thin stringy features parallel to the longitudinal axis of the grains, which grew perpendicular to the boundary of the molten pool. It can also be observed from Figure 4a that the top surface contained plenty of cellular structures and a small amount of columnar structures. The columnar structures were mainly distributed at the boundaries of the molten pool channel, and cellular structures were distributed in the inner of it. This was because the growth direction of cellular-dendritic sub-structures were basically perpendicular to the boundary of the molten pool. In the upper part of the molten pool, the crescent shape boundary of the molten pool was perpendicular to the top surface, resulting in the horizontal distribution of sub-structures [17]. As shown in Figure 4c,e, the cellular structures with the size of about 500 nm had a hexagonal shape. Figure 4b showed columnar sub-structures and melt pools. The cross section (XOZ plane) was dominated by the columnar structures, which was parallel to the BD, consistent with the longitudinal axis direction of the crystal grains. During solidification, heat mostly dissipated along BD in SLM processing. Cellular-dendritic sub-structures also grew along the highest temperature gradient direction, e.g., BD direction. In Figure 4d,f, bundles of these columnar sub-structures in different layers were growing in the same direction, suggesting that the epitaxial growth occurred.

**Figure 5:**
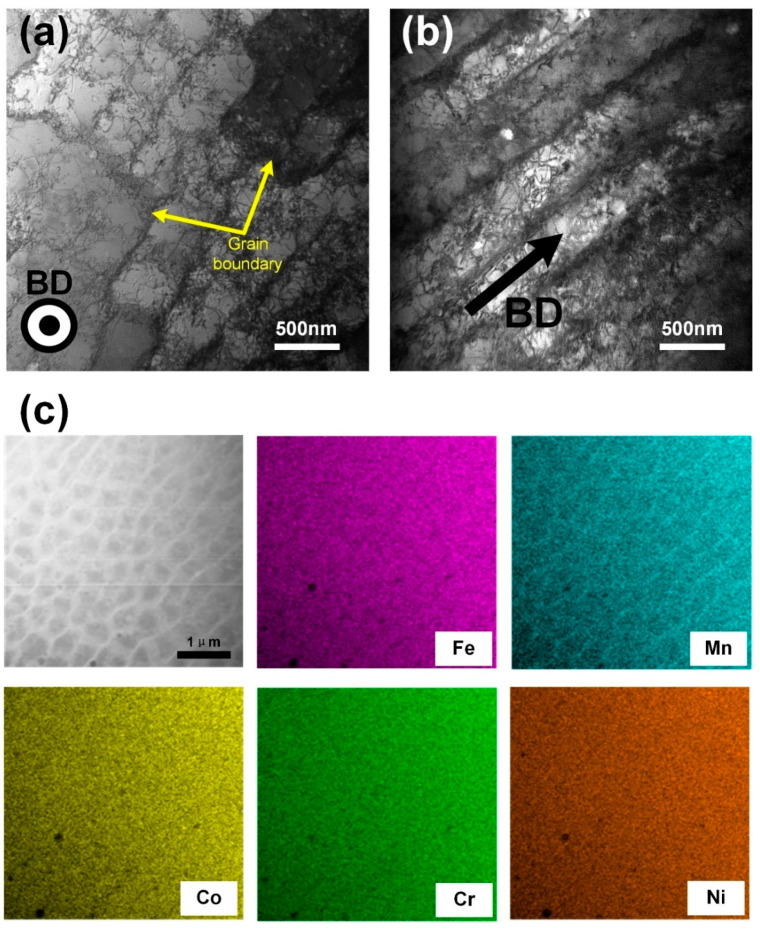
TEM bright field images of the cellular-dendritic sub-structures from (**a**) top surface XOY plane and (**b**) cross section XOZ plane. (**c**) STEM image and elemental distribution maps of the sub-structures.

Figure 5a,b shows the bright-field TEM images of cellular structures and columnar structures in the SLM produced CoCrFeMnNi high-entropy alloy from the top surface and cross section. Grain boundaries, cellular structures, dislocations and cellular walls are shown in Figure 5a. The size of the cellular structures was about 500 nm, which was consistent with the size of the cellular structure observed by SEM (Figure 4e). A small number of dislocations were also visible within the cellular structures. The cellular walls thickness was 80 nm approximately, which were decorated with a high density of dislocations. The size and wall thickness of the cellular structures had been known to be related to the solidification conditions during the SLM processing [18]. As shown in Figure 5c, there was a segregation of Mn along the walls of cellular structures characterized by the compositional mapping. According to the previous report of SLM produced 316L stainless steel [19], although their morphology was similar to dislocation walls, the dislocations were pinned at the walls due to the composition difference between the walls and the matrix. It could be inferred that dislocations formed an obvious enrichment region at the sub-structures’ boundary of SLM processed CoCrFeMnNi high-entropy alloy, so that the strength of the materials was improved.

**Figure 6:**
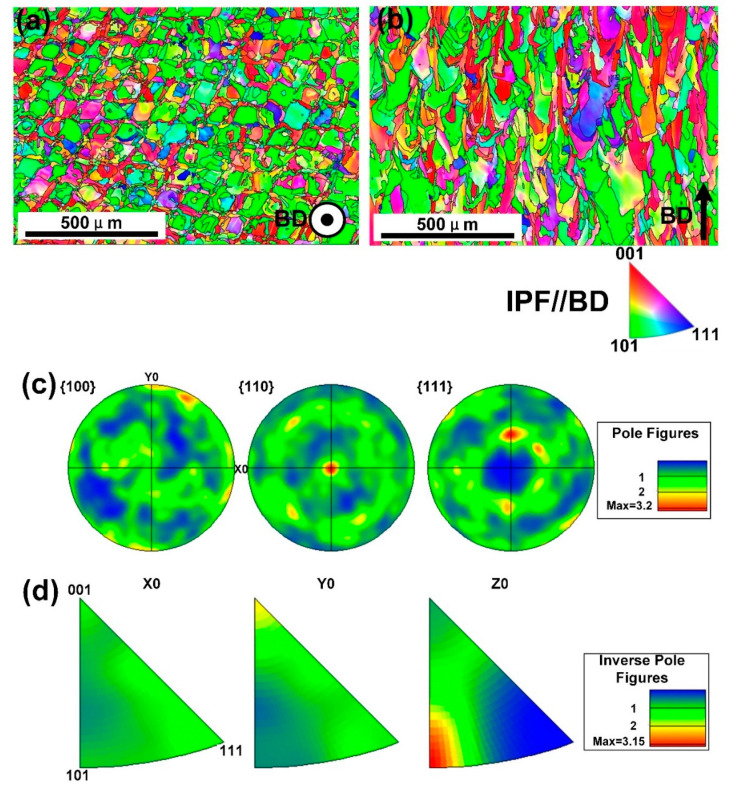
Electron backscatter diffraction (EBSD) orientation maps showing grain morphologies for the SLM processed CoCrFeMnNi samples on the (**a**) top XOY plane and (**b**) side XOZ plane with the EBSD (**c**) pole figure and (**d**) inverse pole figure from XOY plane.

Further characterization was conducted by the EBSD to analyze the texture and the orientation of grains. The EBSD orientation maps, as well as the pole figure and inverse pole figure (IPF) are shown in Figure 6. Figure 6a,b demonstrates the columnar grain morphology in the XOY (perpendicular to BD) and XOZ (parallel to BD) planes colored in the IPF//BD. In the XOZ plane, there was an obvious columnar crystal structure parallel to the BD, which agreed with previous work by Kong D [20]. The formation of columnar crystal structure was largely related to the solidification process in the manufacturing process. During the SLM process, the focused high-energy laser beam irradiated on the fine powder bed, the powder was completely melted prior to form dense materials. The scan was conducted in the same manner layer by layer with a certain scanning strategy, according to the CAD model, and finally formed a dense solid part. The tiny molten pool formed by laser irradiation had an extremely high temperature, causing a very high cooling gradient from the substrate. Under the synergistic effect of the epitaxial growth determined partially by the preferable crystal growth direction, the cross layer columnar structure parallel to the BD was formed. However, observed in the XOY plane, the columnar crystal structure showed a regular checkerboard-like structure, the width of which was under the restriction of the width of the molten pool and the pattern was controlled by the scanning strategy [21,22].

According to Figure 6c,d, the typical EBSD pole figure (PF) and inverse pole figure (IPF) from XOY plane were presented. There was a {110} fiber texture in XOY plane (perpendicular to BD), indicated by the multiple density compared to uniform at the center of the XOY {110} pole figure of the XOY plane. There was also a little {100} fiber texture in the XOY plane, as shown in Figure 6a,d. In general, the samples mainly showed a fiber texture of {110} directions perpendicular to BD. It was known that during the building of SLM specimens each layer would be repeatedly heated by laser energy during the deposition of the following layer, resulting in an actually semi-annealing state in specimens, which would promote the formation of texture. Generally, after the melting and solidification process of SLM, the samples with FCC structure were easy to grow in the <001> preferred growth direction [23]. However, in this case, the preferred growth direction was mainly {110} fiber texture perpendicular to the BD. This could be explained by the crystal characteristics of this particular alloy. During the process each melt-pool had experienced many re-melted and heated events due to the deposition of adjacent tracks and successive layers, so the selection of preferable grains was promoted through growth competition: favorably aligned grains outgrew misaligned ones [8]. In this situation, the grains with {110} fiber texture were the favorably aligned grains. Further studies on the mechanism of CoCrFeMnNi crystal growth in SLM process were needed to explain the phenomenon.

What is more, the grain size also showed differences. For the XOY plane, the average grain size was about 11.9 μm, and for the XOZ plane, the average grain size was about 14.9 μm, which further proved the anisotropic character of the microstructure.

### 3.3. Mechanical Property Evaluation

**Figure 7:**
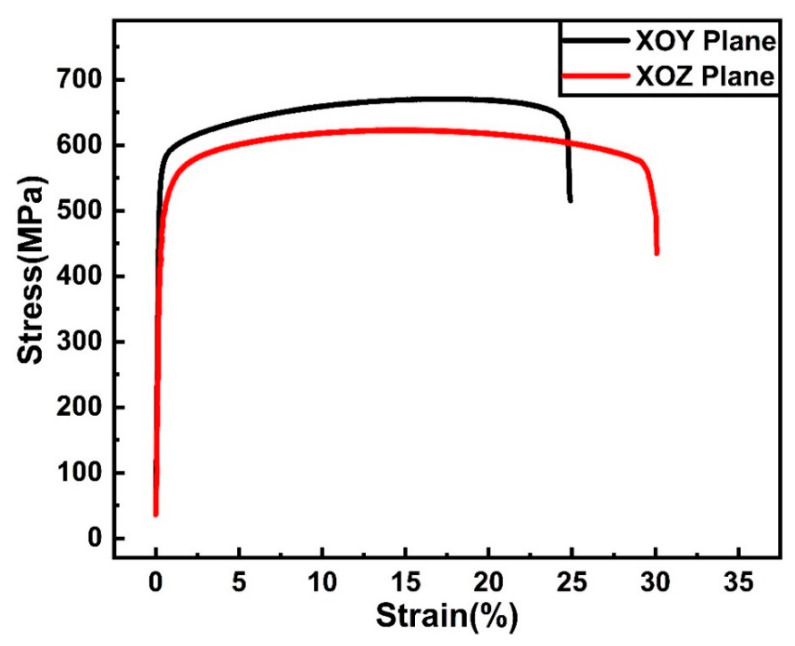
Tensile engineering stress–strain curves of the SLM processed CoCrFeMnNi alloy.

The typical tensile properties of the SLM produced CoCrFeMnNi high-entropy alloy in the parallel (XOZ plane) and perpendicular (XOY plane) to building direction (BD) at room temperature were shown in Figure 7. At least three samples from the same batch using the same parameters were extracted to be tensile tested. The XOY plane samples exhibited excellent mechanical properties with ultimate tensile strength (UTS) of 679.8 ± 12.9 MPa, yield strength (σ_0.2_) of 582.9 ± 10.8 MPa and elongation to failure (ε_f_) of 23.8 ± 1.4%. However, the XOZ plane sample showed slightly lower UTS of 635.9 ± 13.4 MPa, σ_0.2_ of 503.8 ± 10.6 MPa, but better ε_f_ of 31.8 ± 1.5%. As shown in Table 3, the mechanical properties of CoCrFeMnNi high-entropy samples manufactured by SLM were superior to that of casting processed. This could be explained by the fine grain structure formed in SLM processed samples. The formation of the ultrafine cellular-dendritic sub-structures also improved the mechanical properties of the SLM processed alloy.

**Figure 8:**
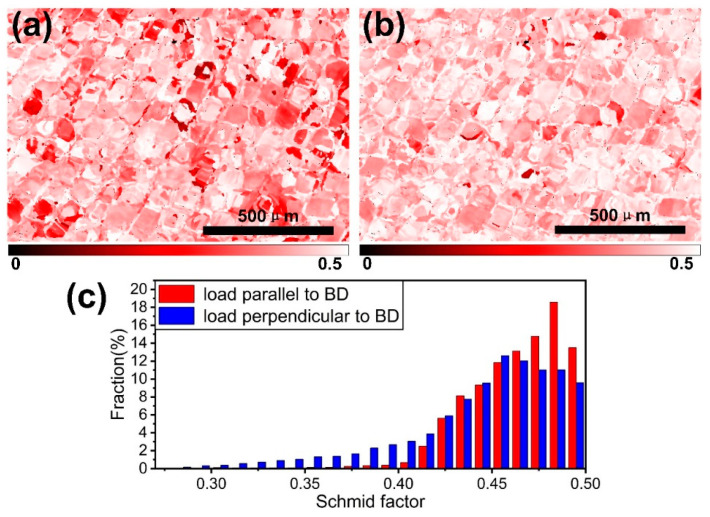
Schmid factor maps for (**a**) loading perpendicular to BD, (**b**) loading parallel to BD and (**c**) corresponding Schmid factor distribution map in (**a**,**b**).

Based on the previous discussion, the SLMed alloys showed a noticeable anisotropic microstructure, which would have affected the properties. The ultimate tensile strength and yield strength of samples from XOZ plane (parallel to BD) were lower, but the elongation was better. Considering the Schmid factor of the crystal, the macroscopic yield strength of the material could be expressed as [25] the following equation:

$$\tau_{c}=\sigma_{s}\cos\lambda \cos\varphi$$

where $\tau_{c}$ was the critical resolved shear stress in slip systems, which only depended on the nature of the material, $\sigma_{s}$ was the yield strength of the material, and cosλcosφ was the Schmid factor (μ). It is only when the shear stress on the sliding plane and the sliding direction reaches a critical value that the metal will undergo plastic deformation. Therefore, the value of the Schmidt factor was reciprocally related to the yield strength. In Figure 8, the Schmid factor distribution in the XOY plane when loading on the different directions were shown. The softest slip system in FCC materials was {111} <110>. Figure 8a,b shows the distribution of Schmidt factors when the loading direction was perpendicular to and parallel to the BD, respectively. The lighter the color, the larger the Schmidt factor and the smaller the yield strength. So, when the loading direction was perpendicular to the BD direction, the yield strength could be larger. Figure 8c shows the frequency distribution of the Schmidt factors of Figure 8a,b. The average value of the Schmidt factor was 0.44 when the load was perpendicular to the BD, and 0.46 when the load was parallel to the BD, suggesting that the yield strength of XOZ plane sample (load parallel to BD) would be lower than that of XOY plane sample.

In addition, the CoCrFeMnNi high-entropy alloy prepared by SLM exhibited a columnar grain structure along the building direction due to its processing characteristics. According to the results of previous studies [26,27,28,29,30], when the load applied along the direction perpendicular to the BD, the yield strength obtained was higher than that of the specimens loaded along the BD, which was due to the difference of grain size in each loading direction. The grain boundary could restrain dislocation movement. The effect of grain size in different direction might be described by the Hall–Petch relation:

$$\sigma_{y}=\sigma_{0}+\frac{K}{\sqrt{d}}$$

where $\sigma_{y}$ was the material yield strength, $\sigma_{0}$ and K were constants related to the material, d was the average grain size. As mentioned above, the XOY and XOZ planes showed different metallographic morphologies, and the grain size was also different. The average grain size in the XOY plane was about 11.9 μm, and the average grain size in the XOZ plane was about 14.9 μm, which made the yield strength of the XOZ plane specimens further lower than that of the XOY plane. There must be some other factors also contributing to the difference. It was speculated that the grain size distribution and crystal orientations might be related, but further investigation was needed to confirm these.

**Figure 9:**
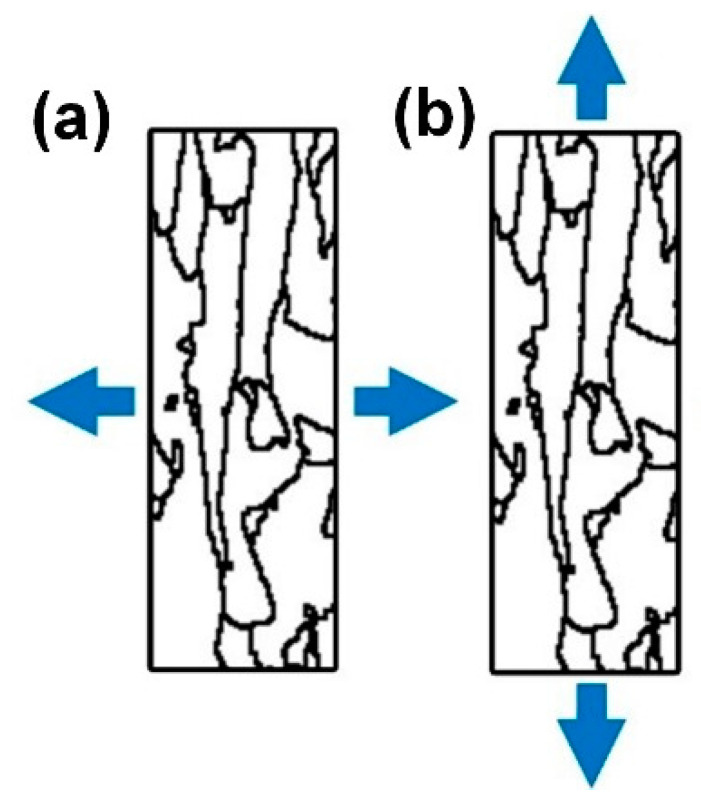
The grain boundaries from the XOZ plane when the load was (**a**) perpendicular to the long axis of columnar grains and (**b**) parallel to the long axis of columnar grains.

The difference of elongation under tension stress was attributed to the different cracking mechanisms between the samples of XOY and XOZ planes. The angles between the tensile load and long-axis direction of columnar crystal played an important role in the elongation. When the tensile direction was perpendicular to the long axis of columnar grains (Figure 9a), many long boundaries of columnar grains were subject to tensile mode I. The existence of dislocation stacking at the grain boundary promoted the formation of microcracks along the columnar grain boundary with the increase of the tension, and finally led to the failure of samples. However, when the tensile direction was parallel to the long axis of columnar grains (Figure 9b), only some short boundaries of columnar grains were subject to tensile action, which meant that it was more difficult for the fracture failure.

**Figure 10:**
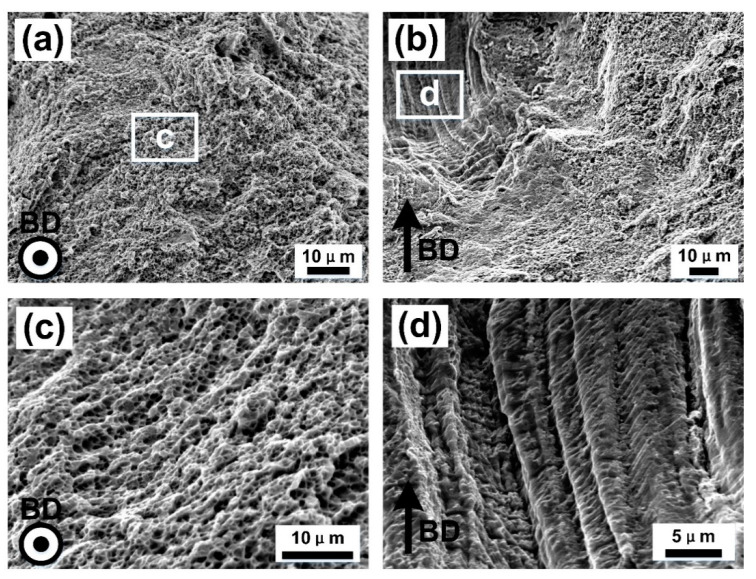
SEM images of fracture surfaces for SLM processed CoCrFeMnNi high-entropy alloy at room temperature. (**a**,**c**) were the fracture morphology of XOZ plane samples, (**b**,**d**) were from XOY plane samples.

In order to further study the fracture mechanism, the fracture surface of CoCrFeMnNi high-entropy samples processed by SLM were observed, as shown in Figure 10. It could be seen that the CoCrFeMnNi high-entropy samples processed in parallel and perpendicular to building direction (BD) show typical ductile fracture characteristics. The small dimples, with the size of about 500 nm—consistent with the size of the cellular structure—were uniformly formed on the whole fracture surface, as shown in Figure 10c, which led to excellent ductility in SLM processed samples. In the late stage of the stress–strain curve in Figure 7, the results showed that the stress decreased with the increase of strain, which proved the existence of plastic deformation in tensile failure. However, In the fracture morphology of XOZ plane, it had not only a large number of dimples, but also a small amount of cleavage surfaces with the cellular structures observed, as shown in Figure 10d, suggesting a conductive brittle hybrid fracture mode. The fracture morphology of the XOY plane sample was the result of the tearing of the grain boundary at the long edge of columnar grains, and a small amount of columnar sub-structures could be observed [30].

### 3.4. Corrosion Property Assessment

**Figure 11:**
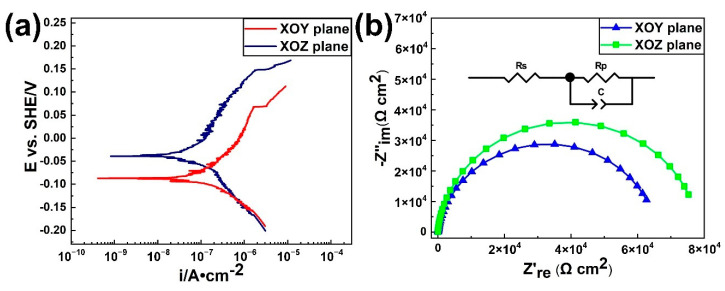
(**a**) the Potentiodynamic polarization curves and (**b**) the EIS results of the XOY plane and XOZ plane for the SLM produced CoCrFeMnNi alloy in the 3.5 wt % NaCl solution at room temperature.

Potentiodynamic polarization curves of the SLM-produced CoCrFeMnNi alloy on the XOY and XOZ planes in the 3.5 wt % NaCl solution were presented in Figure 11a. The electrochemical parameters were summarized in Table 4. The corrosion potential ($E_{corr}$) of XOY plane and XOZ plane were −86.93 mV and −38.04 mV, respectively. In general, the lower $E_{corr}$ meant lower resistance to corrosive reaction [31]. The values of corrosion current density ($I_{corr}$) obtained from XOY and XOZ planes were similar, representing the similar corrosion rate under OCP condition. $E_{pit}$ represented the point at which the corrosion current increases sharply, which meant that pitting pits began to appear in the passive film. Generally, a higher $E_{pit}$ value meant that pitting was more difficult to initiate and it corresponded to a better pitting corrosion resistance [32]. The $E_{pit}$ values of XOY plane and XOZ plane were 70.18 mV and 148.56 mV, respectively, proving that the passive film of XOY plane had worse corrosion resistance against attack of Cl^−^ ion. What is more, the XOZ plane showed a little wider passive range ($\Delta E_{resistance}=E_{pit}-E_{corr}$) of ~186.60 mV compared to the XOY plane (~157.01 mV), which indicated that the passivation film formed on XOZ plane had better stability. The Nyquist plots in Figure 11b were obtained from the electrochemical impedance spectroscopy (EIS) under OCP conditions in 3.5 wt % NaCl solution. Two distinct capacitive arcs were evident for both XOY plane and XOZ plane, with the arc diameter of XOZ plane was slightly larger than that of XOY plane. The diameter of the arc was directly proportional to the polarization resistance of the material, indicating a lower corrosion resistance of the XOY plane compared to the XOZ plane in 3.5 wt % NaCl solution. A modified Randles circuit in Figure 11b was used to fit experimental data to calculate the electrochemical parameters which were listed in Table 4. The simulation results showed that the polarization resistance of XOZ plane was higher than that of XOY plane, while the solution resistance and double-layer capacitance were similar. The electrochemical parameters of as-cast CoCrFeMnNi high-entropy alloy in 3.5 wt % NaCl solution were also listed in Table 4. It could be seen that the corrosion performance of CoCrFeMnNi high-entropy alloy prepared by SLM method was better than that prepared by casting.

**Figure 12:**
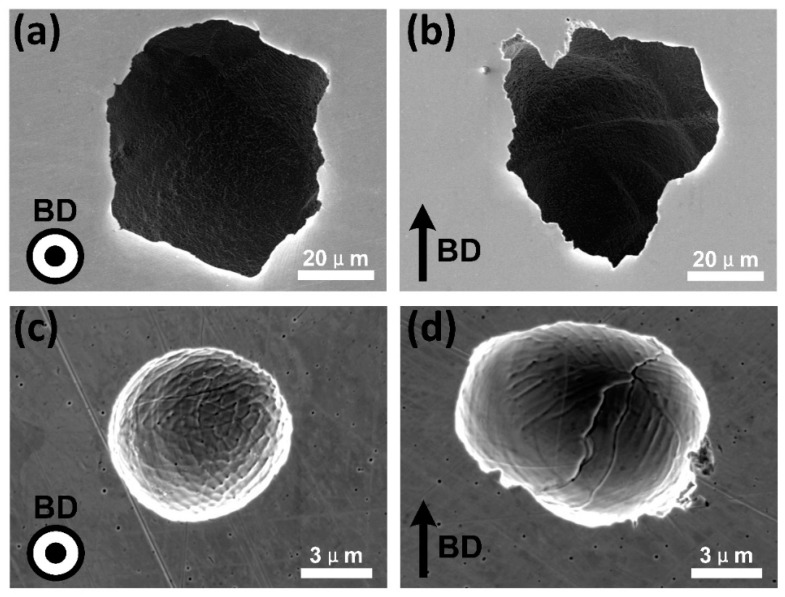
SEM images of the corroded surface for the (**a**,**c**) XOY plane and (**b**,**d**) XOZ plane after polarization test in 3.5 wt % NaCl solution. (**a**,**b**) Were pits with higher corrosion degree, (**c**,**d**) were pits with lower corrosion degree.

After polarization measurement, the corrosion surface of XOY plane and XOZ plane were cleaned ultrasonically and observed by SEM. Figure 12a,b showed the pits with a higher degree of corrosion, (c) and (d) were the pits with a lower degree of corrosion. Pits on both of XOY and XOZ planes exhibited selective corrosion of the wall of cellular-dendritic sub-structures, resulting in the cellular corrosion morphology of XOY plane and the columnar corrosion morphology of XOZ plane. The observed selective attack of the segregated inter-cellular regions could be attributed to the segregation of Mn along the walls of cellular structures. The corrosion potential of Mn was 0.6–1.0 V lower than those of Co, Cr, Fe, and Ni in chloride media which could lead to galvanic coupling effects [33]. What is more, the difference of corrosion properties between the XOY plane and XOZ plane would be on account of the anisotropic microstructure of the cellular-dendritic sub-structures. Specifically, there both existed high interfacial activity between the Mn-rich wall of cell/columnar sub-structures and substrate for XOY and XOZ planes, due to the microgalvanic corrosion occurring in the interface. The total amount of the highly active interface of the XOY plane was much more than that of the XOZ plane (as shown in Figure 4), which greatly accelerated the corrosion reaction [34].

## 4. Conclusions

In this study, the microstructure, mechanical and corrosion anisotropic properties of SLM processed CoCrFeMnNi high-entropy alloy were investigated.

1. The columnar crystal structure with the long axis direction consistent with the BD direction was formed under the extremely high thermal gradient during the SLM process. Within the columnar crystal, cellular-dendritic sub-structures with a size of about 500 nm were found, and their long axis direction were consistent with that of the columnar crystal. The walls of the sub-structures, decorated with high density dislocations, had Mn segregations with the thickness of about 500 nm.
2. The SLMed samples showed fiber textures with dominant {110} directions mixed with some {100} directions perpendicular to the building direction, under the current building strategy.
3. The XOY plane sample (perpendicular to building direction) exhibited higher mechanical properties of ultimate tensile strength and yield strength (σ_0.2_) (UTS of 679.8 ± 12.9 MPa, and σ_0.2_ of 582.9 ± 10.8 MPa), but lower elongation to failure (ε_f_ of 23.8 ± 1.4%), compared to the XOZ plane (parallel to building direction) (UTS of 635.9 ± 13.4 MPa, σ_0.2_ of 503.8 ± 10.6 MPa and ε_f_ of 31.8 ± 1.5%), which was due to the anisotropy of microstructure.
4. In the 3.5 wt % NaCl solution, the XOZ plane exhibited higher $E_{corr}$, $E_{pit}$ and polarization resistance, indicating that the XOZ plane had better corrosion resistance in comparison with the XOY plane. The difference of the corrosion properties between the XOY and XOZ planes were caused by the microstructural features exposed by the tested plane.

Overall, this study showed that SLM produced CoCrFeMnNi alloy could retain the beneficial combination of mechanical properties and corrosion properties, providing a factual basis for the future application of this alloy.

## Figures and Tables

**Table 1:** Chemical Compositions of alloy powder and as-built specimens.

Elements (wt %)	Co	Cr	Fe	Mn	Ni
**Powder**	20.1 ± 0.25	19.5 ± 0.45	20.7 ± 0.5	19.9 ± 0.35	20.8 ± 0.35
**As-built**	20.6 ± 0.35	18.7 ± 0.8	21.0 ± 0.6	18.3 ± 0.25	21.3 ± 0.35

**Table 2:** Diffraction peak intensity ratio.

Intensity Ratio	Original Powder	XOY Plane	XOZ Plane
$f_{200}$	0.37	1.03	0.15
$f_{220}$	0.27	1.98	0.11

**Table 3:** Tensile properties of CoCrFeMnNi alloy at room temperature.

Processing	σ0.2 (MPa)	UTS (MPa)	εf (%)	Ref.
SLM produced, XOY plane	582.9 ± 10.8	679.8 ± 12.9	23.8 ± 1.4	This work
SLM produced, XOZ plane	503.8 ± 10.6	635.9 ± 13.4	31.8 ± 1.5	This work
Cast	275	475	51	[24]

**Table 4:** Electrochemical and equivalent circuit parameters of the XOY plane and XOZ plane for the SLM produced CoCrFeMnNi alloy in the 3.5 wt % NaCl solution at room temperature.

Corrosion Parameter	XOY Plane	XOZ Plane	As-Cast
$E_{corr}\;\left(\mathrm{mV}\right)$	−86.83	−38.04	−457
$I_{corr}\;\left(\mu A/{\mathrm{cm}}^{2}\right)$	0.19	0.1	1.123
$E_{pit}\;\left(\mathrm{mV}\right)$	70.18	148.56	-
$\Delta E_{resistance}\;\left(\mathrm{mV}\right)$	157.01	186.60	-
Polarization Resistance, $R_{p}\;\left(\mathrm{ohm}/{\mathrm{cm}}^{2}\right)$	65,903	71,466	12,700
Solution Resistance, $R_{s}\;\left(\mathrm{ohm}/{\mathrm{cm}}^{2}\right)$	5.982	5.988	5.2
Double Layer Capacitance, $C\;\left(\mu F/{\mathrm{cm}}^{2}\right)$	35.29	32.47	19.37
	This work	This work	[14]
